# M2 macrophages-derived exosomes regulate osteoclast differentiation by the CSF2/TNF-α axis

**DOI:** 10.1186/s12903-023-03842-x

**Published:** 2024-01-18

**Authors:** Yue Zhou, Guangyao Hu

**Affiliations:** Department of Stomatology, Affiliated Hospital of Beihua University, Building 7, Hongda Lanwan Community, Risheng Road, High-tech Zone, Jilin City, Jilin Province 132011 China

**Keywords:** Exosomes, M2 macrophages, Osteoclast differentiation, CSF2, TNF-α

## Abstract

**Background:**

Osteoclast-mediated bone resorption cause bone loss in several bone diseases. Exosomes have been reported to regulate osteoclast differentiation. M2-polarized macrophages exhibit anti-inflammatory activity. This study aimed to explore the effect of exosomes from M2 polarized macrophages (M2-exos) on osteoclastogenesis and molecular mechanisms.

**Methods:**

M2-exos were isolated from IL-4-induced Raw264.7 cells (M2 macrophages) and used to treat osteoclasts (RANKL-induced Raw264.7 cells). Osteoclast differentiation was visualized using tartrate resistant acid phosphatase staining. Quantitative real-time PCR (qPCR) was conducted to measure the levels of osteoclastogenesis-related genes. The underlying mechanisms of M2-exos were evaluated using qPCR and western blotting.

**Results:**

M2-exos suppressed osteoclast differentiation induced by RANKL. Additionally, CSF2 was highly expressed in M2 macrophages, and knockdown of CSF2 further enhanced the effects of M2-exos on osteoclast differentiation. Moreover, CSF2 positively regulated TNF-α signaling, which inhibition promoted differentiation of M2-exo-treated osteoclasts.

**Conclusion:**

M2-exos inhibited RANKL-induced osteoclast differentiation by downregulating the CSF2 expression through inactivating the TNF-α signaling, suggesting the potential application of exosomes in bone disease therapy.

**Supplementary Information:**

The online version contains supplementary material available at 10.1186/s12903-023-03842-x.

## Introduction

Bone provides mechanical support for body and locomotion. Continuous bone remodeling is the key to maintaining bone homeostasis [1]. The balance of bone resorption and bone formation ensures normal bone remodeling without changes in bone mass and quality [2]. Once the bone remodeling process is disordered, it leads to the occurrence of various bone diseases, such as osteoporosis, osteopetrosis, and periodontitis [3, 4]. Bone formation is mediated by osteoblasts, while bone resorption is mediated by osteoclasts. Osteoclasts, originate from the monocyte/macrophage hematopoietic lineage, regulate bone resorption, and are associated with bone erosion and bone remodeling in bone diseases [5, 6]. Osteoclasts can adhere to bone matrix and degrade them by secreting acids as well as lytic enzymes [7]. RANKL and CSF1 induce osteoclast lineage gene expression to foster osteoclast maturation, ultimately leading to bone remodeling. Hence, inhibiting osteopenia by inhibiting osteoclast differentiation may prevent bone loss in bone disease including periodontitis.

Exosomes are extracellular microvesicles ranging from 30 to 150 nm in diameter, derived from almost all kinds of cells and body fluids [8, 9]. They contain a multitude of substances, such as proteins, nucleic acid, and lipids, which regulate cell-to-cell communication [10]. Due to the stability and the active substances they release, exosomes serve as regulatory mediators in the development of many diseases, including periodontitis [11]. Macrophages are innate immune cells that can activate or resolve inflammation [12]. They were classified according to the M1/M2 dichotomy. When macrophages polarize into an M1 phenotype, they release pro-inflammatory cytokines. Conversely, macrophages polarizing into an M2 phenotype exhibit anti-inflammatory function [13]. However, whether polarized macrophages participate in osteoclast differentiation through the secretion of exosomes is not well understood.

In this study, we aimed to investigate whether exosomes released from M2 macrophages (M2-exos) regulate osteoclast differentiation and the underlying mechanism. We constructed a RANKL-induced cell model and treated it with M2-exos to explore osteoclast differentiation. This study will provide theoretical support for exosomes as candidates for the treatment of bone diseases.

## Materials and methods

### Cell culture

Mouse mononuclear macrophages Raw264.7 (ATCC) were cultured in DMEM containing 10% FBS (Procell, Wuhan) and 1% penicillin–streptomycin (Procell) at 37 °C with 5% CO_2_ and 95% atmosphere.

### M2 macrophages induction

Raw264.7 cells were cultured in DMEM supplemented with 10% exosome-free FBS (Absin, Shanghai). Then, the Raw264.7 cells were treated with 20 ng/mL of interleukin (IL)-4 for 24 h to induce M2 macrophages [14].

### Exosome separation

The M2-exos were isolated from the culture medium of M2 macrophages using ultracentrifugation as previously described [15]. The medium was centrifugated in turn according to the following conditions: 300 ×g for 15 min, 3,000 ×g for 15 min, and 20,000 ×g for 70 min. After filtering using the filters (0.22 μm pore size). The supernatant was further centrifugated at 120,000 ×g for 70 min and washed with PBS, Finally, the exosomes were suspended in PBS and stored at -80 °C until use.

### Exosome identification

The characteristics of M2-exos including morphology, particle size, and markers were examined by transmission electron microscopy (TEM), nanoparticle tracking analysis (NTA), and western blotting, respectively. For TEM, M2-exos were added to carbon-precoated copper mesh (200 mesh) and stained with 1% uranyl acetate for 60 s. The morphology was visualized using a Tecnai Spirit TEM (FEI, Czech). For NTA, M2-exos were diluted using PBS and assessed using the Zetasizer Nano ZS90 instrument (Malvern, UK).

### Osteoclast induction and exosome treatment

Raw264.7 cells were seeded into 96-well plates and treated with 50 ng/mL RANKL recombinant protein (MedChemExpress, Monmouth Junction) for 4 days. The induction solution was replaced every 2 days.

The isolated M2-exos (50 µg/mL) were used to treat osteoclasts in the presence of RANKL [16].

### Cell transfection

Small interfering RNAs (si-CSF2#1 and si-CSF2#2) and the corresponding negative control (si-NC) were synthesized by Genepharma (Shanghai). M2 macrophages were transfected with siRNAs using lipofectamine 2000 reagent following the protocol of the manufacturer. After 48 h of transfection, the efficiency was detected.

### Adalimumab treatment

To inhibit TNF-α levels, 10 µg/mL of Adalimumab (ADA; Anti-Human TNF-α; MedChemExpress) was added to the cell culture medium to incubate with cells for 2 h before RANKL treatment [17].

### Tartrate resistant acid phosphatase (TRAP) staining assay

Osteoclast differentiation was measured using a TRAP staining kit (BestBio biotechnology, Shanghai) following the manufacturer’s protocol. Raw264.7 cells were fixed with stationary liquid from the kit for 3 min and washed using water for 1 min. Then, the samples were incubated with the working solution in the dark for 60 min. After re-staining with hematoxylin, the stained cells were observed.

### Quantitative real-time PCR (qPCR)

Total RNA was extracted from M2 macrophages and Raw264.7 cells using the TRIzol reagent (Invitrogen, Carlsbad). Reverse transcription was conducted using the M-MuLV first chain cDNA synthesize kit (Sangon Biotech, Shanghai) with the conditions of 42 °C for 45 min and 70 °C for 10 min (stop reaction). An SGExcel FastSYBR qPCR mixture (Sangon) was used for quantitative PCR on the ABI 7500 system with the conditions of 95 °C for 3 min, 40 cycles of 95 °C for 5 s and 60 °C for 20 s. Relative mRNA expression was determined using the 2^−ΔΔCt^ method. GAPDH was used as the normalization.

### Western blotting

Raw264.7 cells and M2 macrophages were lysed using the RIPA lysis buffer on ice. Proteins were extracted from M2-exos using the exosome protein extraction kit (EZBioscience, Roseville). Afterwards, an equal amount of proteins were run to separate using the 10% SDS-PAGE and transferred to PVDF membranes (Millipore, Billerica). The membranes were blocked using 5% skim milk and incubated the following primary antibodies at 4 °C overnight. The antibodies (Abcam, Cambridge) including anti-TSG101 (ab125011, 1/1000), anti-CD9 (ab263019, 1/1000), anti-TNF-α (ab183218, 1/1000), anti-TRAF2 (ab126758, 1/1000), anti-FADD (ab124812, 1/1000), and anti-GAPDH (ab9485, 1/2500). A secondary antibody (ab97051, 1/10,000) was continued to incubate with the membranes at room temperature for 1 h. The bands were visualized using an ECL western blotting substrate kit (Abcam).

### Bioinformatics

The protein-protein interaction network of CSF2 was predicted using the STRING database (https://cn.string-db.org/).

### Data analysis

The experiments were conducted at least in triplicate independently. The results were analyzed using the GraphPad Prism 8 software and presented as the mean ± standard deviation. Comparisons were analyzed by Student’s t-test or one-way analysis of variance. P value < 0.05 was considered statistically significant.

## Results

### Exosomes are successfully isolated from M2 macrophages

**Fig. 1:**
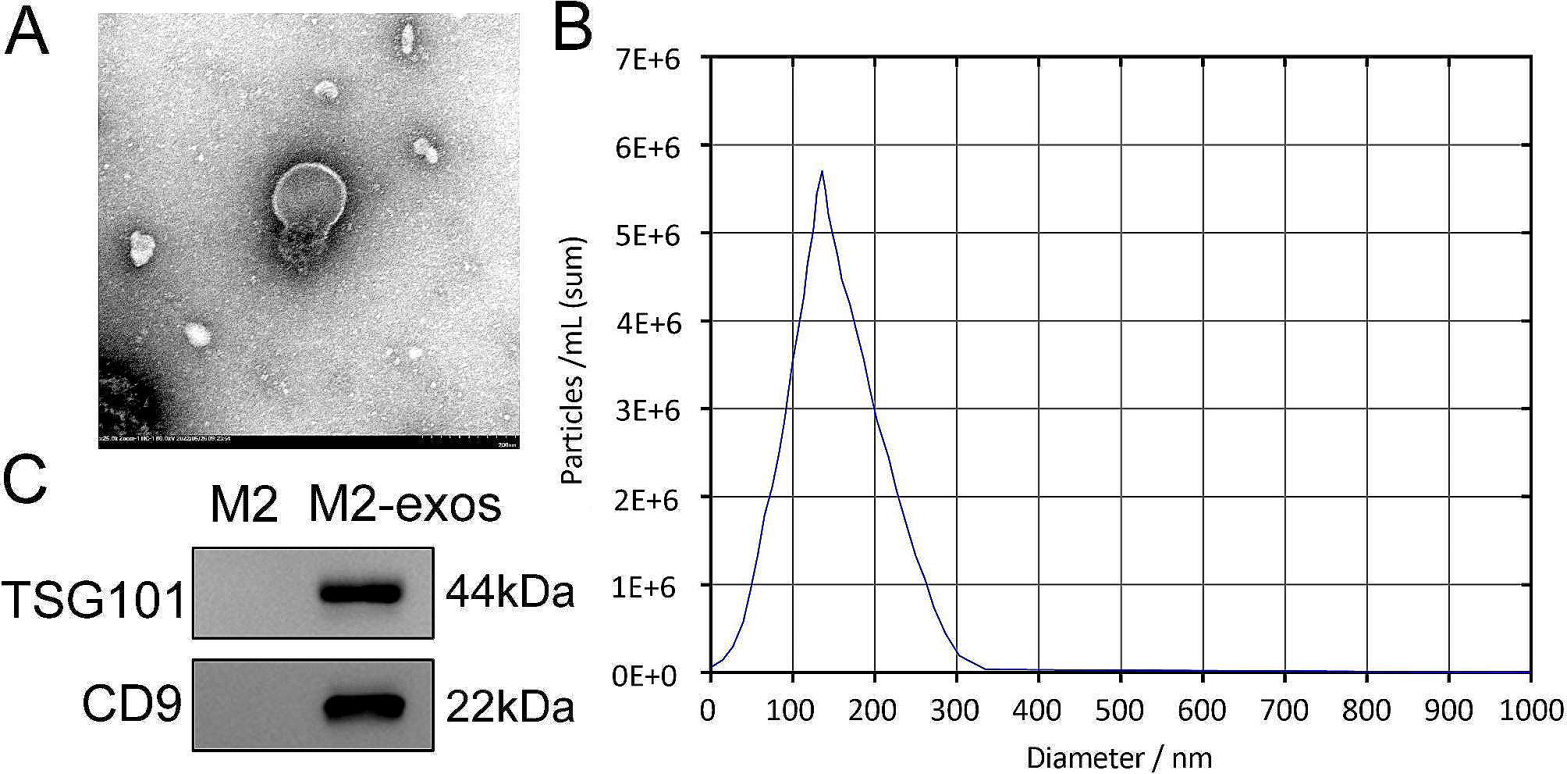
Exosomes are successfully isolated from M2 macrophages. (**A**) Exosome morphology was observed by TEM. (**B**) The particle size of exosomes was identified using NTA. (**C**) Exosome surface markers TSG101 and CD9 were examined using western blotting

To obtain M2-exos, Raw264.7 macrophages were treated with IL-4 to induce an M2 phenotype. Then, exosomes were collected from the culture medium and authenticated. We observed that M2-exos were spherical nanoparticles with clear membranes using TEM (Fig. 1A). The particle size of exosomes mainly concentrated in 100–200 nm (Fig. 1B). Besides, TSG101 and CD9 proteins were expressed in M2-exos, while the macrophages did not show these proteins (Fig. 1C). Thus, we concluded that we successfully isolated exosomes from M2 macrophages.

### M2-exos suppress RANKL-induced osteoclast differentiation

**Fig. 2:**
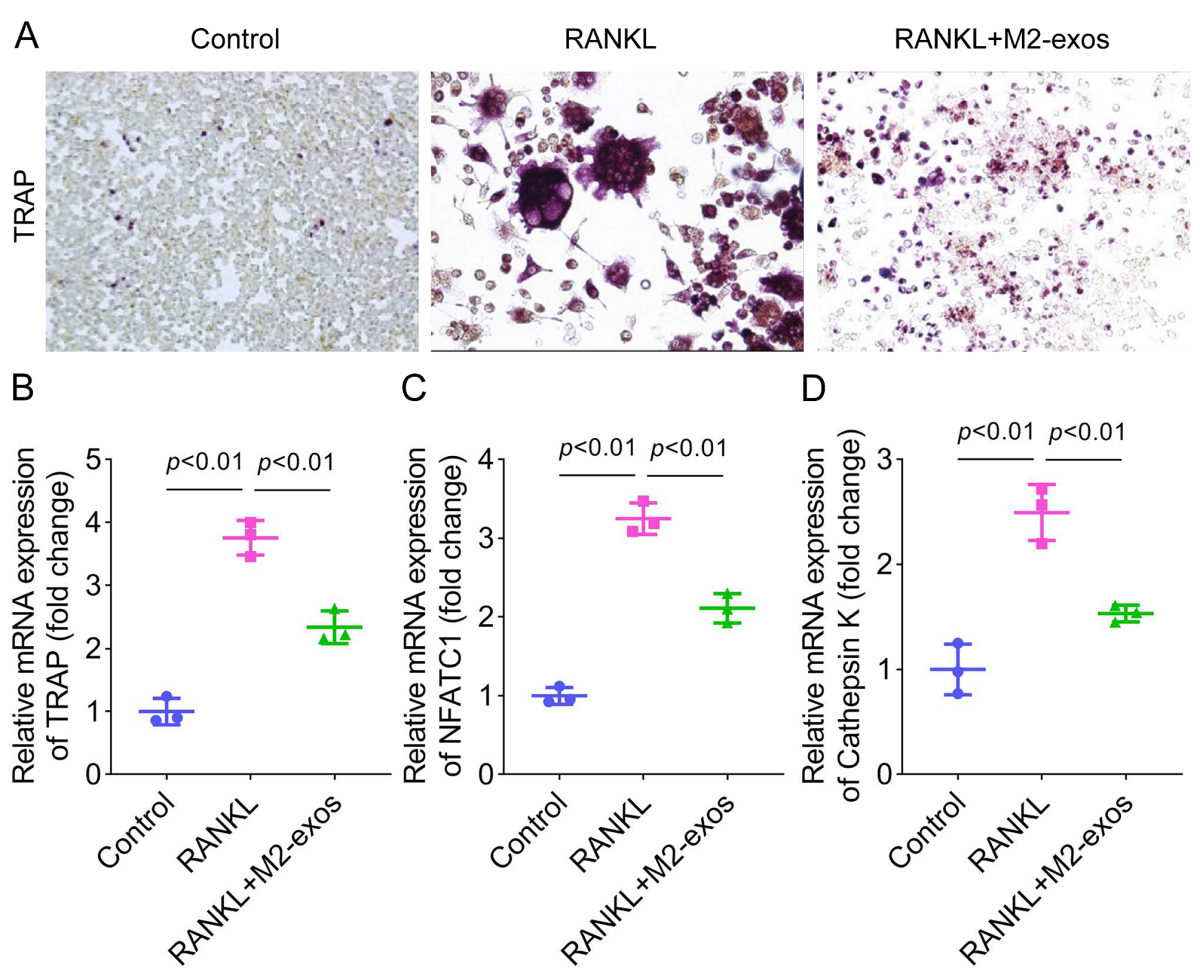
M2-exos suppress RANKL-induced osteoclast differentiation. (**A**) Raw264.7 cells were treated with RANKL and M2-exos, and osteoclast-like cells were observed using the TRAP staining assay. (**B**) TRAP, (**C**) NFATC1, and (**D**) Cathepsin K mRNA expression was detected using qPCR. **P < 0.01 vs. the control group. ##P < 0.01 vs. the RANKL group

Subsequently, we assessed the effects of M2-exos on osteoclast differentiation. Raw264.7 cells were exposed to RANKL to induce osteoclastogenesis, and then treated with M2-exos. As shown in Fig. 2A, RANKL increased TRAP-stained cells and represented typical osteoclast morphology, and M2-exos treatment reduced these osteoclast-like cells. Additionally, the mRNA expression of osteoclast markers including TRAP, NFATC1, and Cathepsin K was elevated after RANKL treatment, while M2-exos partly counteracted this elevation (Fig. 2B and 2D).

### CSF2 is highly expressed in M2-exos and is required for osteoclastogenesis

**Fig. 3:**
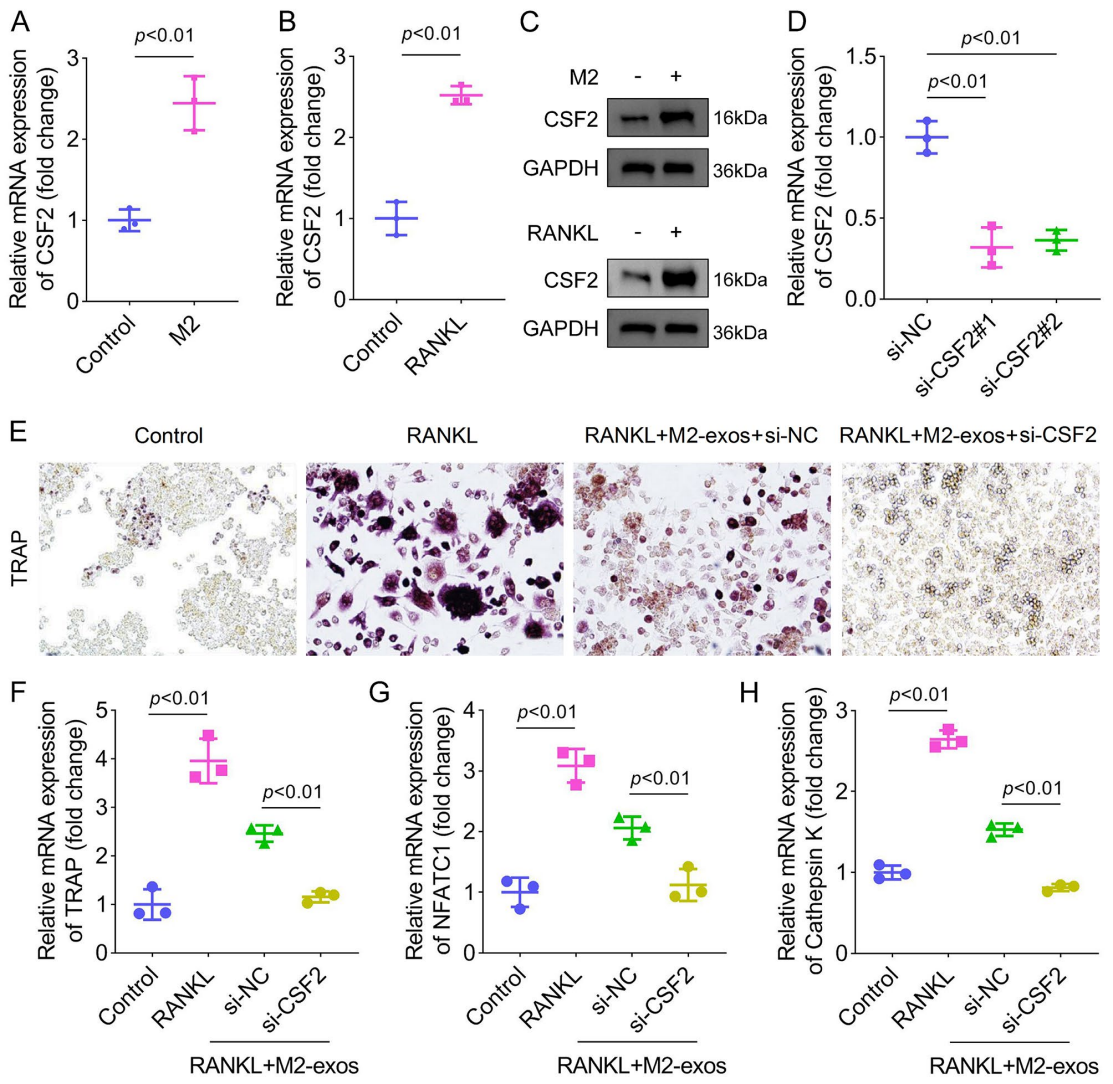
CSF2 is highly expressed in M2-exos and is required for osteoclastogenesis. (**A**) CSF2 mRNA expression in M2 macrophages. (**B**) CSF2 mRNA expression in RANKL-treated Raw264.7 cells. (**C**) Protein levels of CSF2 in M2 macrophages and RANKL-induced Raw264.7 cells. (**D**) The efficiency following si-NC, si-CSF2#1, and si-CSF2#2 transfection. (**E**) Osteoclast differentiation was analyzed using the TRAP staining assay. Osteoclast markers (**F**) TRAP, (**G**) NFATC1, and (**H**) Cathepsin K were detected by qPCR. **P < 0.01 vs. the control or si-NC groups. ##P < 0.01 vs. the RANKL + M2-exos + si-NC group

A previous study has revealed that M2-exos carry the CSF2 gene [18]. Therefore, we speculated that CSF2 is involved in regulating osteoclast differentiation by M2-exos. The levels of CSF2 were upregulated in M2 macrophages (Fig. 3A and 3C). In addition, RANKL induced the increase of CSF2 levels (Fig. 3B and 3C). Thus, we transfected si-CSF2 into M2 macrophages to downregulate CSF2 levels (Fig. 3D). The osteoclast differentiation was subsequently evaluated. The results showed that interfering with CSF2 reduced TRAP-stained intensity in osteoclasts treated with M2-exos (Fig. 3E). The mRNA expression of TRAP, NFATC1, and Cathepsin K in RANKL-induced cells was decreased by M2-exos, which was further decreased by CSF2 depletion (Fig. 3F and 3H). The results indicated that exosomal CSF2 derived from M2 macrophages inhibited osteoclast differentiation.

### CSF2 positively regulates TNF-α signaling

**Fig. 4:**
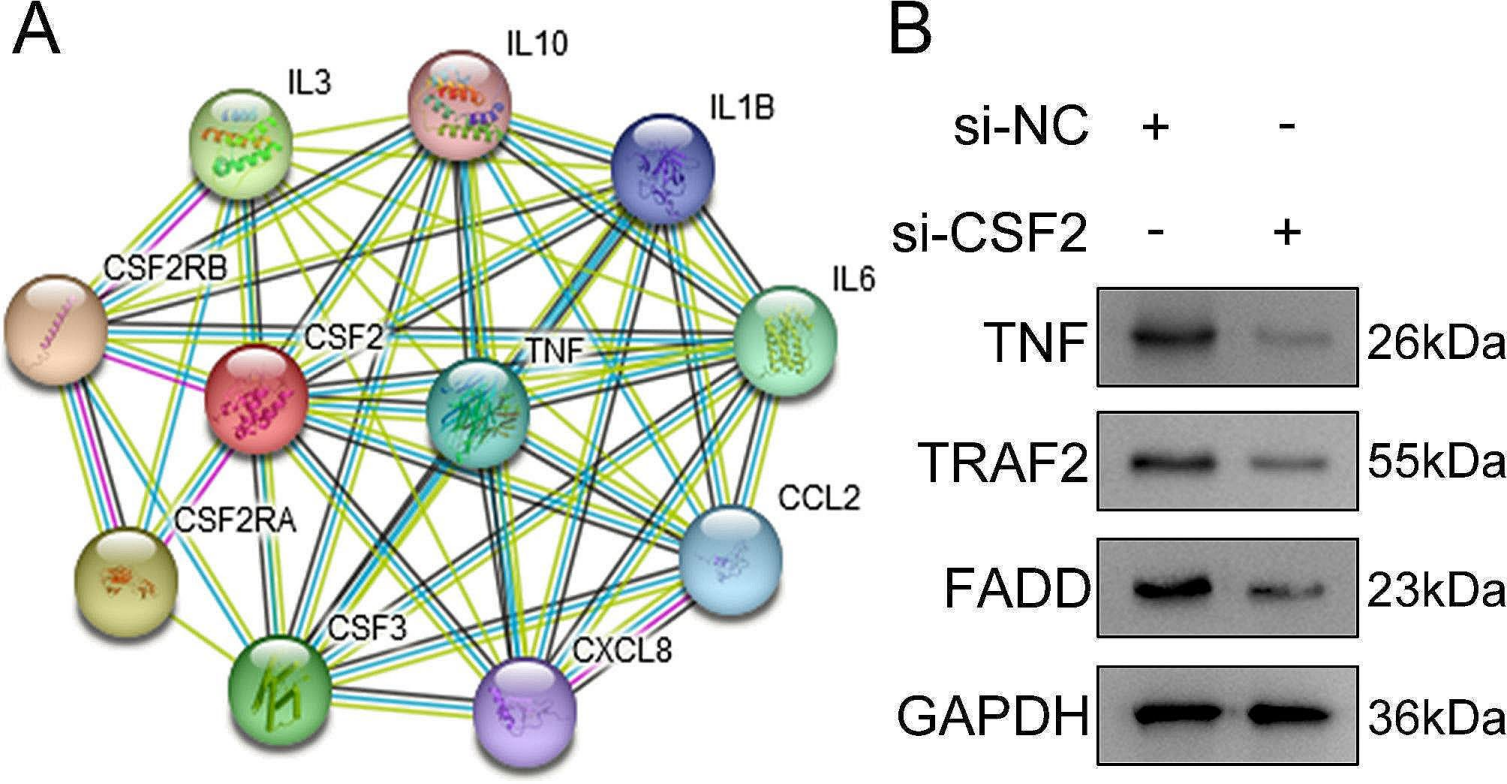
CSF2 positively regulates TNF-α levels. (**A**) The interaction between CSF2 and TNF was predicted using the STRING database. (**B**) The protein levels of TNF, TRAF2, and FADD were detected using western blotting following CSF2 knockdown

To explore the underlying mechanism of CSF2, the interacted proteins with CSF2 were predicted, and we found that TNF could interact with CSF2 (Fig. 4A). Then, CSF2 knockdown downregulated the protein levels of TNF, TRAF2, and FADD (Fig. 4B). The results indicated that interference with CSF2 inhibited the TNF-α signaling.

### M2-exos suppress osteoclast differentiation by regulating TNF-α

**Fig. 5:**
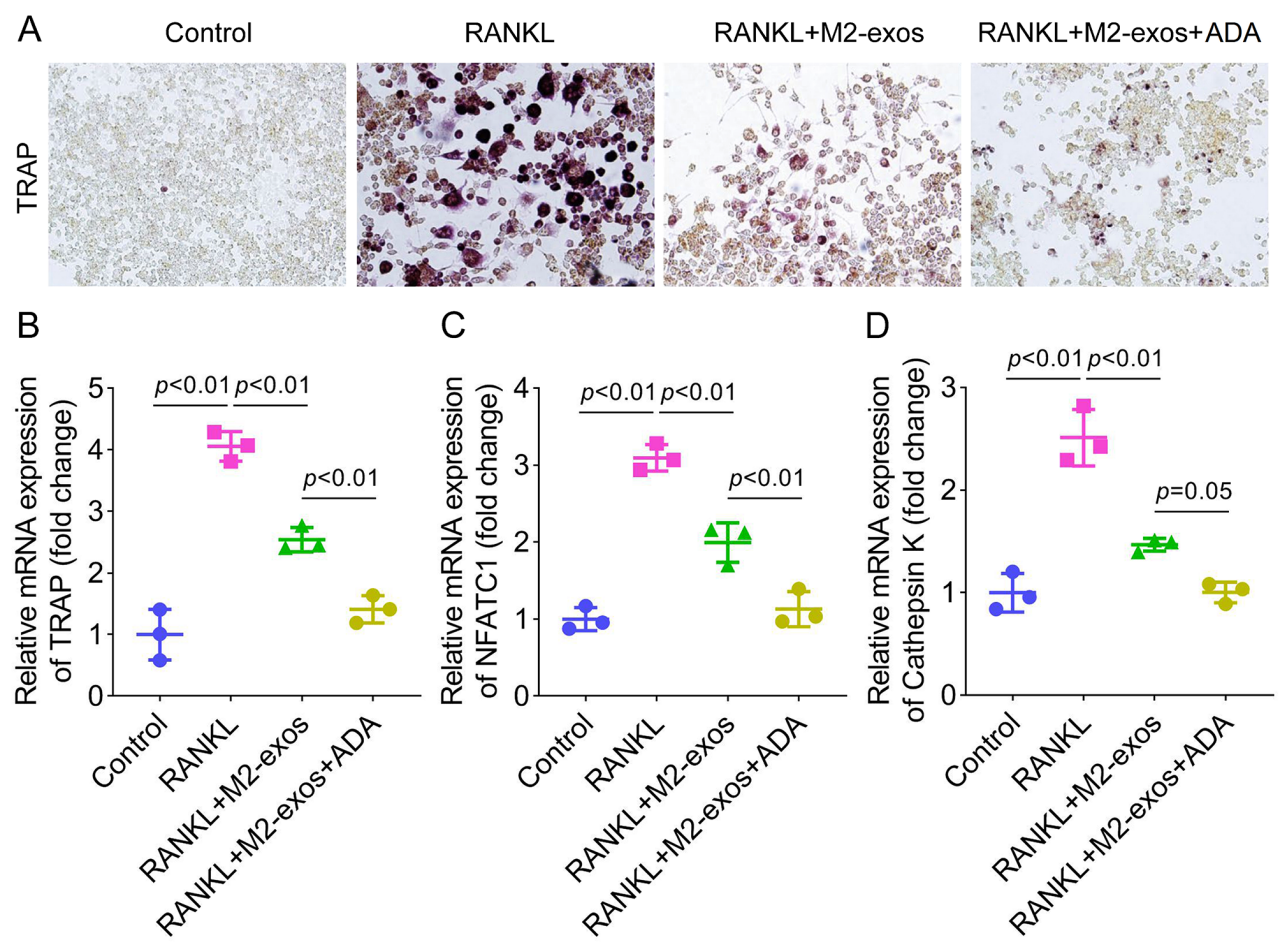
M2-exos suppress osteoclast differentiation by regulating TNF-α. (**A**) The osteoclasts were treated with M2-exos and ADA, and the osteoclast-like cells were stained using the TRAP assay. The mRNA expression of (**B**) TRAP, (**C**) NFATC1, and (**D**) Cathepsin K was examined by qPCR. **P < 0.01 vs. the control group. ##P < 0.01 vs. the RANKL group. &&P < 0.01 vs. the RANKL + M2-exos group

To investigate the effects of TNF-α on osteoclastogenesis, ADA was added to treat osteoclasts to inhibit TNF-α. The results illustrated that ADA further reduced TRAP-stained cells, compared with M2-exos (Fig. 5A). Besides, the increased TRAP, NFATC1, and Cathepsin K induced by RANKL were downregulated by M2-exos, which were further downregulated by ADA treatment (Fig. 5B and 5D). To sum up, M2-exos inhibited osteoclast differentiation by inhibiting TNF-α.

## Discussion

Osteoclast-mediated bone resorption is still a difficult problem that cannot be solved during bone diseases clinical treatment. However, the regulatory mechanism bone resorption is complex and largely unelucidated. For example, multiple factors are involved in the progression of periodontitis, such as pathogenic oral microorganisms, immune activation, oxidative stress, and aberrant bone metabolism [19–21]. Of note, the release of proinflammatory cytokines promotes bone resorption, leading to periodontitis-related bone destruction [22]. Thus, it is important to investigate the regulation of osteoclast differentiation. Exosomes derived from several cells, such as mesenchymal stem cells [23], periodontal ligament fibroblasts [24], and periodontal ligament stem cells (PDLSCs) [25], that are linked with the inflammatory state [11]. M2-exos have been reported to have anti-inflammatory effects and may be used to treat rheumatoid arthritis [26]. Moreover, M2-exos play an important role in bone formation and resorption balance. Previous studies have shown that M2-exos increase pro-inflammatory cytokines and suppress osteoblast differentiation of bone marrow mesenchymal stem cells and PDLSCs [27, 28]. In addition, Chen et al. [29] have reported that M2-exos impede bone marrow-derived macrophage osteoclast formation via the IL-10/IL-10R pathway, thereby reducing alveolar bone resorption. However, the effects of M2-exos on osteoclastogenesis of mononuclear macrophages remain unknown. Here, we isolated M2-exos to treat RANKL-stimulated Raw264.7 cells. The results first indicated that M2-exos decreased TRAP-stained cells and downregulated osteoclast differentiation makers in RANKL-treated Raw264.7 cells. The findings suggest that M2-exos inhibit osteoclast differentiation, then may decelerate the progression of bone diseases.

CSF2, also known as granulocyte/macrophage CSF (GM-CSF), is an inflammation-associated gene. CSF2 has pro-inflammatory effects and is involved in the pathogenesis of various diseases, such as rheumatoid arthritis, cancer, and metabolic diseases [30]. It has been reported that CSF2 is related to bone remodeling and overgrowth [31, 32]; however, how it affects osteoclast differentiation has not been studied. Moreover, CSF2 regulates the biological behaviors of neutrophils and macrophages, including cell survival, growth, and differentiation [30]. Magnusson et al. [33] have found that CSF2 increases osteoclasts. Li et al. [34] have revealed that the inhibition of bone marrow monocyte-derived osteoclast differentiation by SPIO@15HA is accompanied by increasing CSF2 expression. M2-exos contain CSF2 [18], thus, we identified whether exosomal CSF2 is involved in osteoclast differentiation regulated by M2-exos. We observed that CSF2 was highly expressed in M2 macrophages and RANKL increased CSF2 expression in Raw264.7 cells. Then, we knockdown CSF2 levels in M2 macrophages. Silencing of CSF2 further inhibited osteoclast differentiation, thereby enhancing M2-exo function. Taken together, M2 macrophage exosomes inhibited osteoclast differentiation by secreting the CSF2 gene.

Furthermore, we predicted CSF2-related proteins. We found that TNF is related to CSF2. TNF-α is an immune inflammatory mediator that is associated with chronic inflammation [35]. TRAF2 and FADD are important membranes in TNF-α signaling adapters [36]. TNF-α plays a critical role in the bone metabolism process. Studies have reported that TNF-α regulates osteoblast and osteoclast differentiation [37, 38]. TNF-α inhibits the activity of osteoblasts at differentiation stages, and stimulates the proliferation and differentiation of osteoclasts [39]. Moreover, TNF-α induces osteoclast differentiation in several diseases, such as rheumatoid arthritis, tooth movement, and periodontitis [39–41]. Therefore, we explored whether M2-exo can affect TNF-α during osteoclast differentiation. In the present study, ADA treatment further suppressed M2-exo-mediated osteoclast differentiation. Based on these findings, M2-exos suppressed RANKL-induced osteoclastogenesis by the CSF2/TNF-α axis. A study has revealed that stimulation of TNF-α induces macrophage polarization into an anti-inflammatory M2 phenotype [42]. Hence, we considered that M2-exos suppressed osteoclast differentiation by inhibiting TNF-α, and TNF-α inhibition may reduce M2 macrophages and decrease their secretion of exosomes, forming a feedback loop.

The major limitation of this study is that we only explored the effects of M2-exos on osteoclast differentiation through in vitro experiments, but did not study the effects of M2-exos on bone diseases such as osteoporosis and periodontitis in vivo, which will further studied in our future work.

In conclusion, M2-polarized macrophage-derived exosomes inhibited RANKL-induced osteoclast differentiation by downregulating CSF2 and inhibiting TNF-α. This study provides a theoretical basis and a new regulatory mechanism for exosomes in the treatment of bone diseases.

### Electronic supplementary material

Below is the link to the electronic supplementary material.

**Supplementary Material 1:** Supplementary information-original western blot images

## Data Availability

The datasets used and/or analysed during the current study are available from the corresponding author on reasonable request.
