# Supplementary material for: M2 macrophages-derived exosomes regulate osteoclast differentiation by the CSF2/TNF-α axis
Source: BMC Oral Health. 2024 Jan 18;24:107. doi: 10.1186/s12903-023-03842-x (PMC10795354; doi:10.1186/s12903-023-03842-x)
Supplement: Supplementary file 1 — Supplementary Material 1: Supplementary information-original western blot images [file 12903_2023_3842_MOESM1_ESM.docx]

| Fig 1 | M2 | M2-exos |
| --- | --- | --- |
| TSG101 | 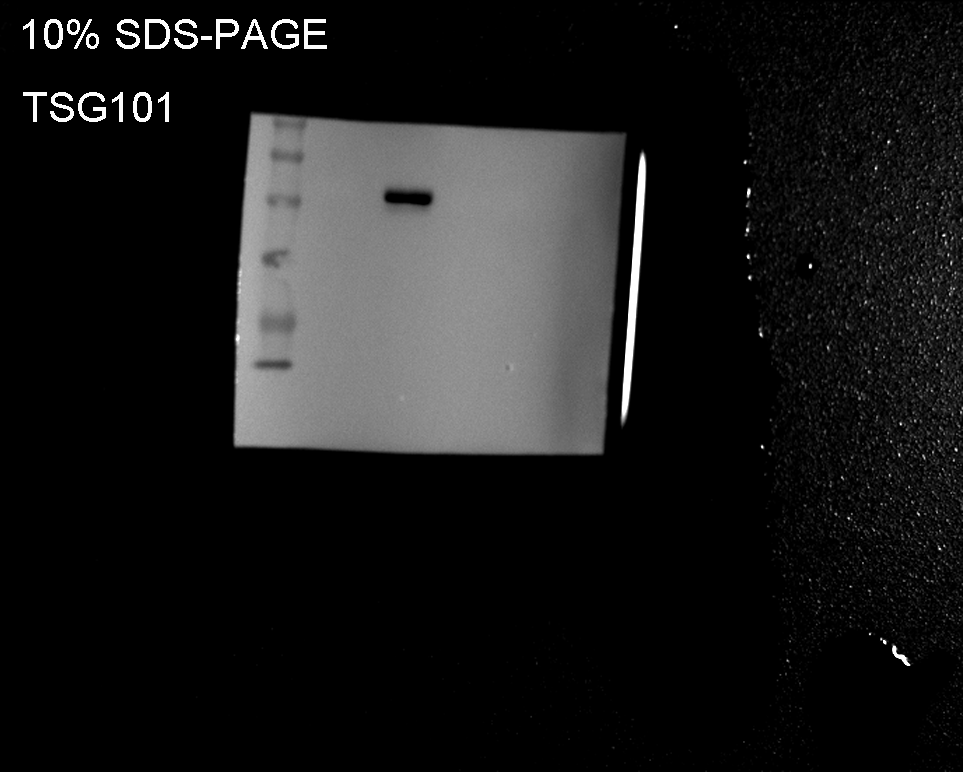 | |
| CD9 | 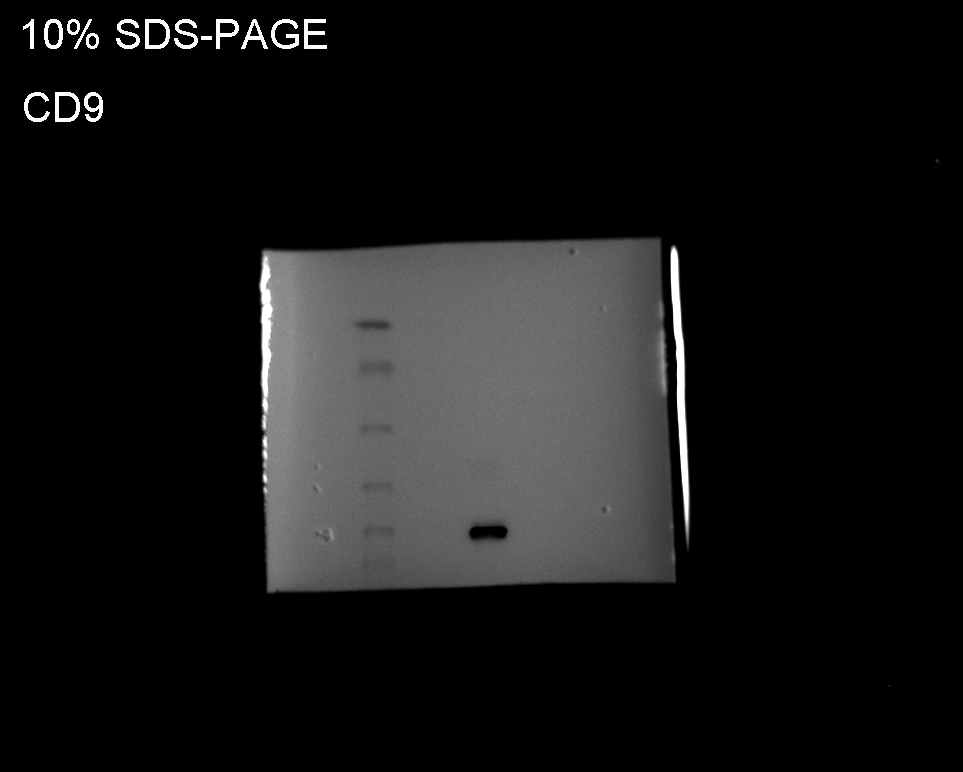 | |

Supplementary information-original western blot images

Figure S1. The original western blot images for TSG101 and CD9 in M2 macrophages and M2-exos that is shown in Fig. 1C.

| Fig 3 | Control | M2 |
| --- | --- | --- |
| CSF2 | 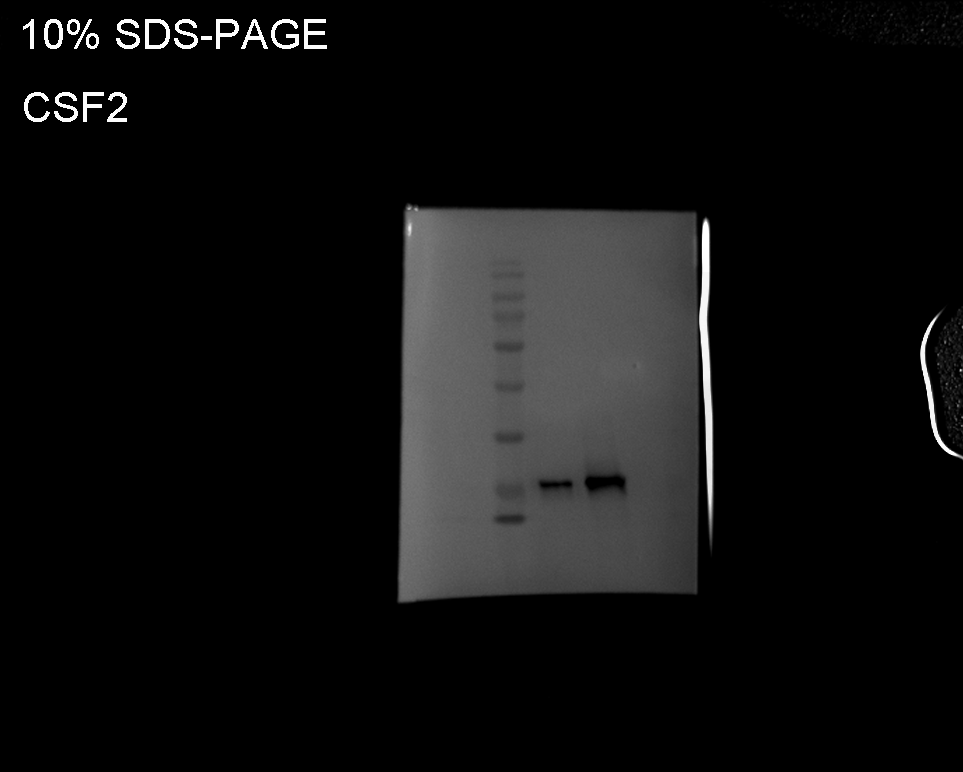 | |
| GAPDH | 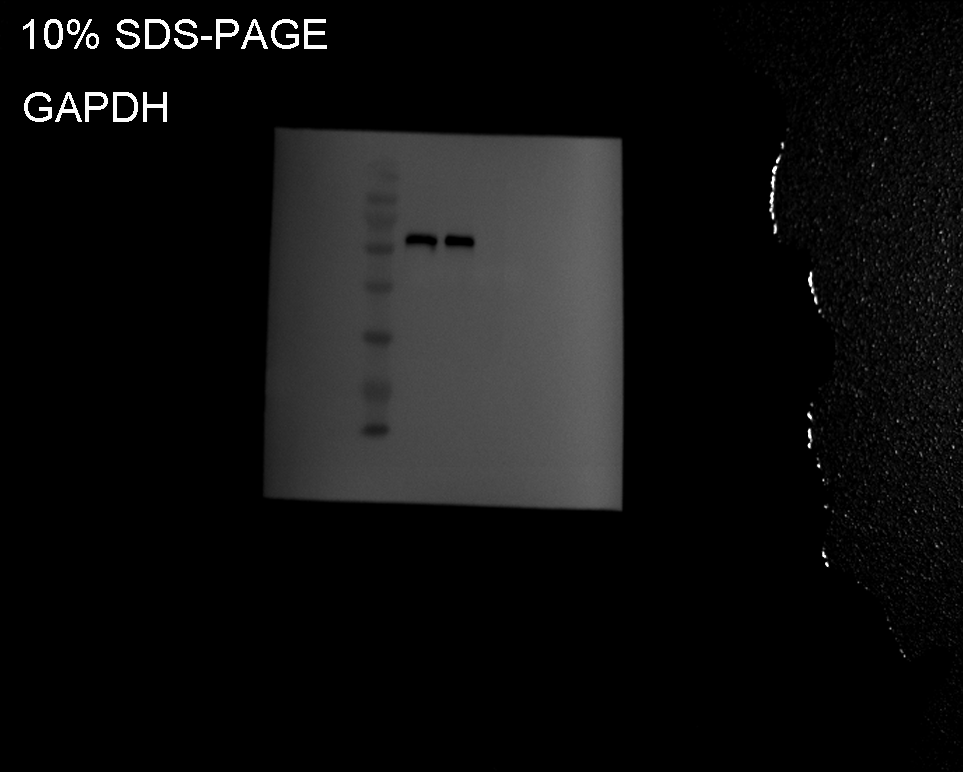 | |

Figure S2. The original western blot images for CSF2 and GAPDH in Raw264.7 cells (control) and M2 macrophages that is shown in Fig. 3C.

| Fig 3 | Control | RANKL |
| --- | --- | --- |
| CSF2 | 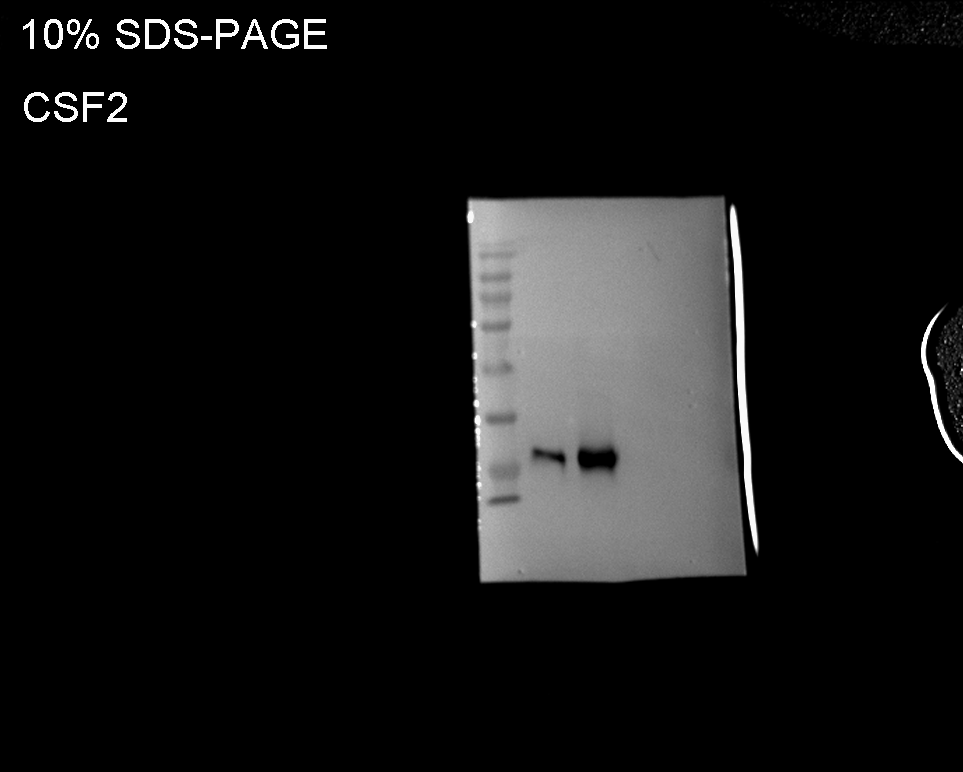 | |
| GAPDH | 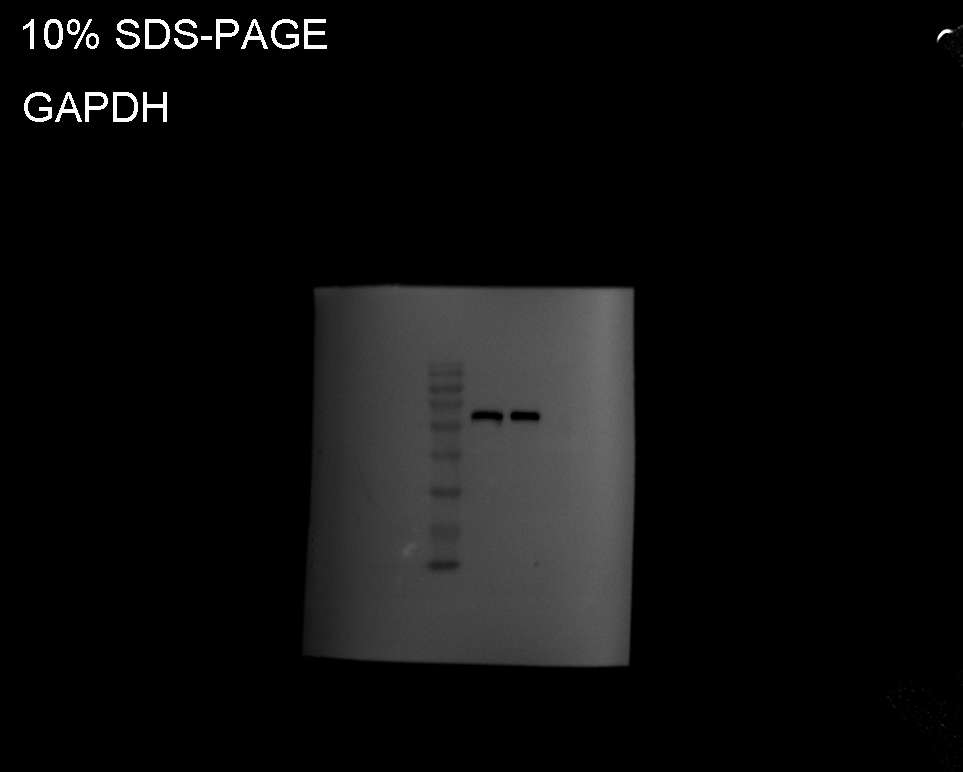 | |

Figure S3. The original western blot images for CSF2 and GAPDH in Raw264.7 cells (control) and RANKL-induced Raw264.7 cells that is shown in Fig. 3C.

| Fig 4 | Si-NC | Si-CSF2 |
| --- | --- | --- |
| TNF | 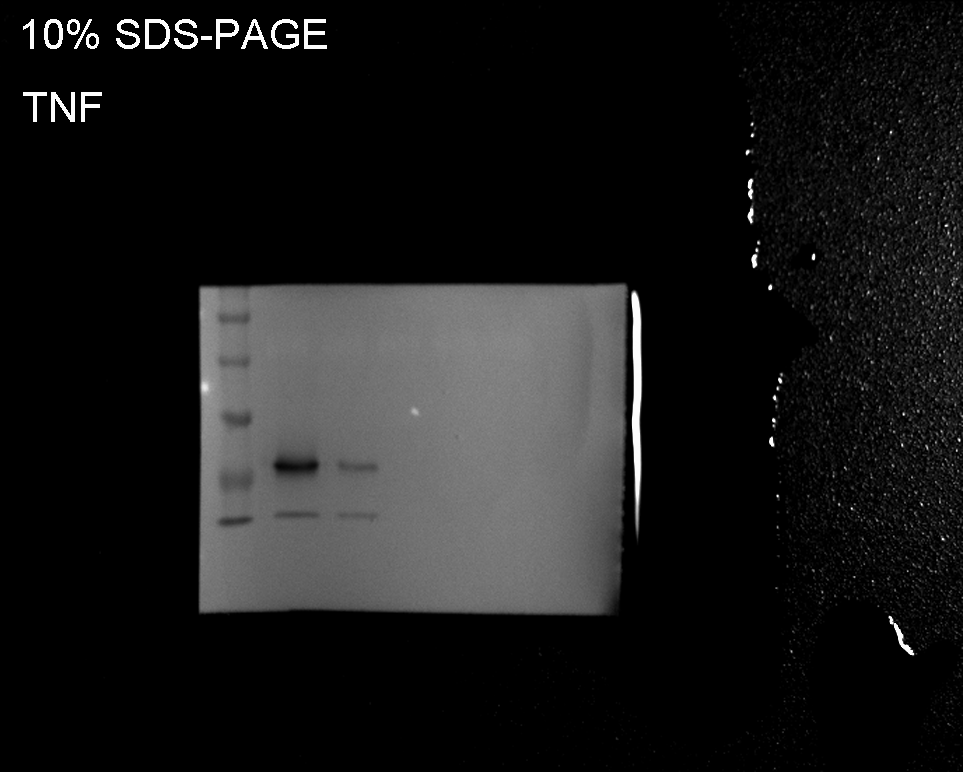 | |
| TRAF2 | 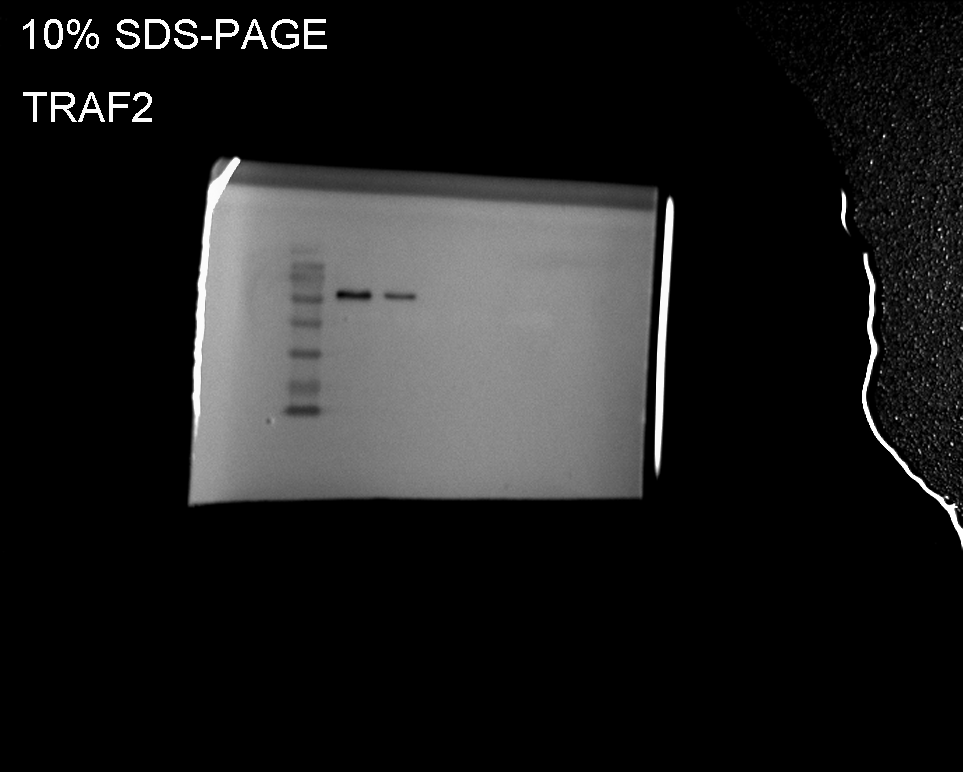 | |
| FADD | 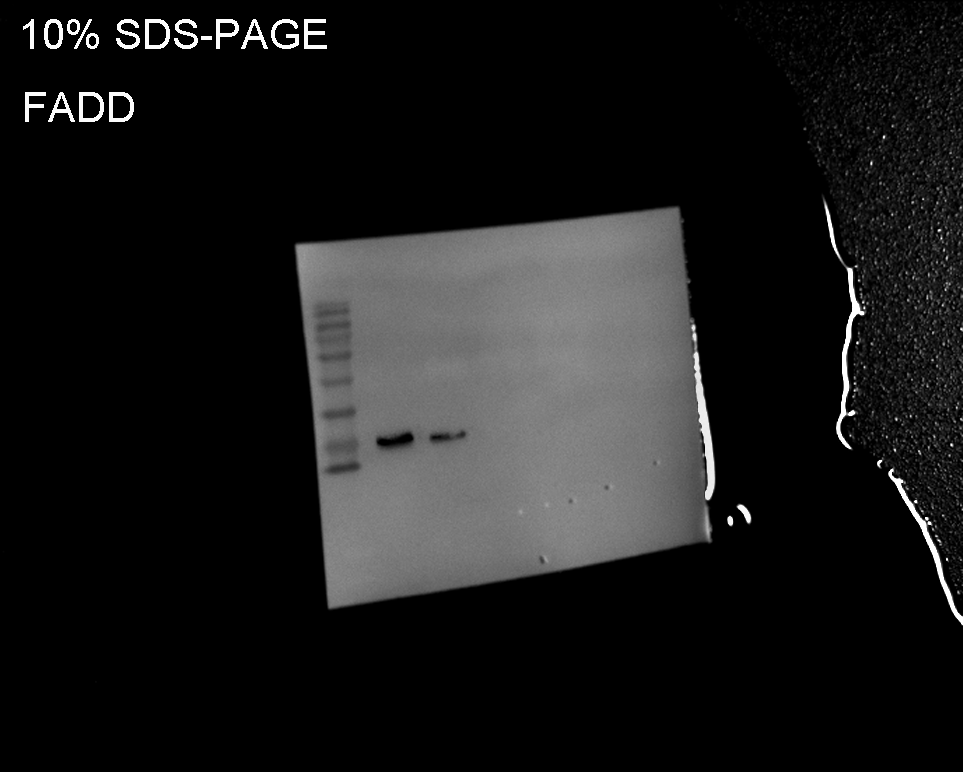 | |
| GAPDH | 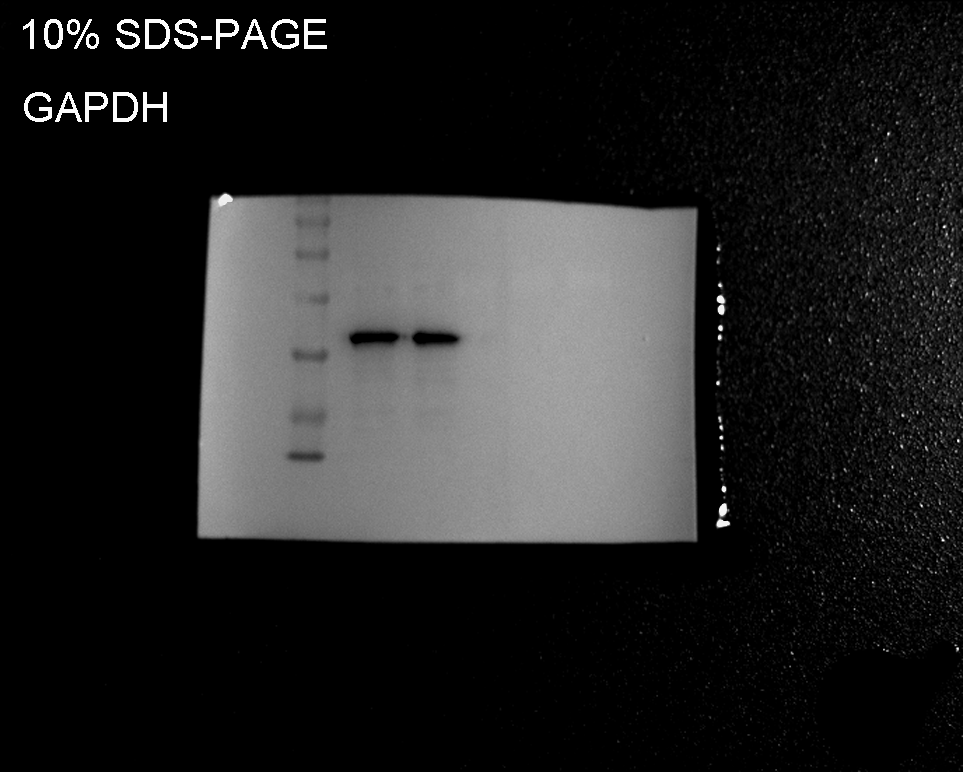 | |

Figure S4. The original western blot images for TNF, TRAF2, FADD, and GAPDH after si-NC or si-CSF2 transfection that is shown in Fig. 4B.
